# Alex's Lemonade Stand Foundation squeezes in hope for childhood cancer: an interview with Liz and Jay Scott

**DOI:** 10.1242/dmm.052967

**Published:** 2026-05-05

**Authors:** Liz Scott, Jay Scott, Alex's Lemonade Stand Foundation

**Affiliations:** Alex's Lemonade Stand Foundation for Childhood Cancer, Wynnewood, PA 19096, USA

**Figure:**
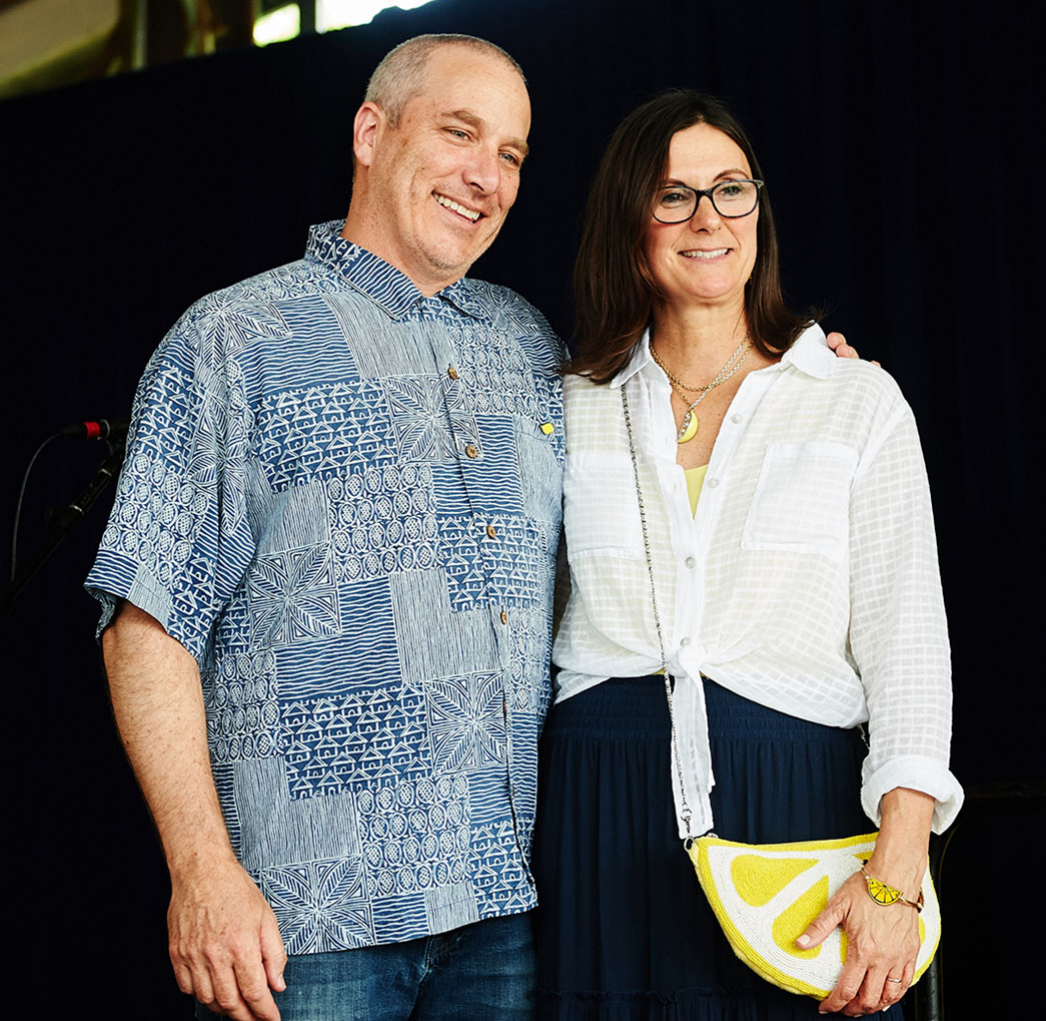
**Jay (left) and Liz (right) Scott.** Photo credit: Philip Gabriel Photography.

Individually, childhood cancers are rare; but, collectively, they impact a huge number of children and their families. Globally, an estimated 400,000 children and adolescents are diagnosed with cancer every year (Steliarova-Foucher et al., 2017), and it is the leading cause of death by disease in children after infancy in the USA (Siegel et al., 2024). The cause of childhood cancer is often elusive, and the most common types are leukaemia, brain tumours, lymphomas and solid tumours, such as neuroblastoma. Efficacy of treatment hinges on early and accurate diagnosis, and each cancer requires a specific treatment regimen. Standard therapies include chemotherapy, surgery and/or radiotherapy, but more tailored, less toxic treatment options are limited. Despite the astounding impact of childhood cancer, it remains underfunded and understudied.

Alexandra (Alex) Scott was diagnosed with neuroblastoma just before her first birthday. Despite this devastating diagnosis, in 2000, at the age of 4, she set up a lemonade stand to raise money to find a cure and help other children with cancer. She raised $2000 at her initial lemonade stand, which encouraged people to set up similar stands all over the world. In the years that followed, Alex continued her fundraising activities while battling neuroblastoma, but she sadly passed away in 2004 at the age of 8. Alex and the people she inspired had already raised $1 million to help find a cure for childhood cancer. In 2005, Alex's parents, Liz and Jay Scott, set up Alex's Lemonade Stand Foundation (ALSF; Box 1), and her legacy has continued to grow. ALSF is now the largest independent childhood cancer charity in the USA. Here, Liz and Jay reflect on how their extraordinary daughter has impacted the lives of so many. They also discuss the unique challenges in childhood cancer research and how they maximise their influence to support patients and their families, as well as researchers searching for a cure.

Box 1. Alex's Lemonade Stand Foundation Alex's Lemonade Stand Foundation (ALSF) is the largest independent childhood cancer charity in the USA, having funded more than 1500 research projects at nearly 150 institutions across North America and Europe. Since Alex's first lemonade stand, they have raised more than $300 million that goes towards supporting families affected by childhood cancer as well as research.

They fund research in two core programmes: Early Career Research and Accelerator Programmes. Both programmes have several grants, ranging from funds for early independent investigators setting up their laboratories to those for more established researchers who are aiming to translate their research to the clinic to improve the care of patients with paediatric cancer. Another area of funding is the Crazy 8 Initiative, where ALSF identified eight prevailing challenges in paediatric cancer that could be solved with innovative and rigorous approaches. ALSF also encourages dissemination of results and cross-disciplinary collaboration to propel innovative research by bringing researchers together at their various research summits and by hosting Childhood Cancer Speaker Series. Although each cancer is rare, these researchers can learn from each other to instil tangible progress.

In 2017, ALSF set up The Childhood Cancer Data Lab, which generates tools and databases and provides training to help researchers maximise the potential of big data generated by modern research methods. Another invaluable scientific resource established by ALSF is the Childhood Cancer Repository. This repository collates validated cell lines and patient-derived xenografts from childhood cancers and shares these with scientists to enable fundamental research.

Alongside ALSF's extensive funding for research, they provide vital support for the children and families living with cancer. Their Travel For Care Programme helps families pay for travel and accommodation to allow all children to access the treatments they need that may be further afield. As ALSF is built upon lived experience, they recognise the importance of providing opportunities that are not simply for practical reasons. For instance, they offer free professional photography for families to capture precious memories and organise family camps. ALSF also supports siblings of children with cancer through their SuperSibs programme. SuperSibs receive special mailings, online activities and school support, and they can get involved with the Sibling Ambassador Programme, which empowers them to advocate for children and families affected by cancer.

## Can you briefly outline ALSF's main goals?

**Liz:** Our foundation is primarily dedicated to funding research to find new cures for all types of childhood cancer. We also support families in many different ways, and we empower people everywhere to get involved and be a part of solving the problems of childhood cancer with us.

## Childhood cancer is a broad disease area; how do you manage the variety of issues and how do you focus your research funding efforts?

**Jay:** Over the years, we've just looked for gaps and tried to fill them. We didn't try to just create something because we felt like it. We tried to create things that there was a need for in the community. So that's how we developed our grant programme. Slowly over time, as we saw needs, we would open up new types of grants and do the same with the services that we provide to families.

**Liz:** As we were raising more money and growing, we recognised that we could put more money in, and we could fill larger gaps. There are still some really large gaps that we think will be pretty expensive to try to fill, so we constantly have a wish list of areas we'd like to fund. As we're raising more money, we're able to put it to use pretty urgently and quickly, because, as parents who have lived through it, we understand that there's a huge sense of urgency every single day to find new cures for kids and to support families.

That's where the voice of the parents is louder to me than the voice of the physicians or researchers. It's one thing if you hear it from one person, but if you hear it repeatedly, over and over again, you just can't deny the fact that there are needs that aren't being met.

## How do patient advocates get involved and help to set research priorities in the foundation?

**Liz:** Well, it's twofold. For the research gaps, we look to the research community to talk to us. We will send out surveys, or we have meetings where they come together. We have a Scientific Advisory Board that we also go to. We combine that with what we hear from families about the challenges they still have across the board, in terms of getting new treatments and the availability and access to the treatments. For our family services, we survey families and social workers in hospitals, but families also come to us every day looking for resources. So, we are tracking those requests to learn about the needs that are coming up again and again in the community of families, which we might be able to solve for them.

We also have ambassadors who we meet with quarterly. That's where these needs come up, and they ask if we've thought about funding different areas. I think survivorship is a good example that comes up quite a bit. In this area, families we talked to feel like there are some pretty big gaps, but then when you talk to the physician, sometimes they don't necessarily see those gaps. This is probably because they're maybe not working with the survivors. Those are the problems that I find really interesting, when the community of survivors and parents are saying there are needs that aren't being met, and there seems to be a disconnect. The reality is that there are so many survivors, and there are a lot of needs in the survivor community. That's where the voice of the parents is louder to me than the voice of the physicians or researchers. It's one thing if you hear it from one person, but if you hear it repeatedly, over and over again, you just can't deny the fact that there are needs that aren't being met.

Listening to the parents and the survivors, as they get older, is what the best physicians do. I think researchers who aren't seeing patients and are working only in the lab need to take their viewpoint into account to think about the impact their work can have. If you approach your science from that perspective, it might change the way you're making decisions, the aims of your work and how you design your experiments.

## Are the researchers that are funded by ALSF generally open to suggestions from you and the advocates?

**Liz:** We have a pretty collaborative process. When we have an idea and we think there's a need, we will sometimes informally bounce it off researchers in the community. We will also more formally bring it to our Scientific Advisory Board. Usually through these conversations, we all get to the place where we recognise that there is a need and agree upon how we might approach it in a way that is smart, makes good use of the money and could have results. It's not a combative relationship. It's a highly collaborative relationship between us, as we bring ideas from the patient community to our researchers and our scientific advisors, and they help us figure out how we can solve that problem. Of course, you might have ten different scientists, especially physician scientists, and they might have different ideas about what things should be the priority. But generally, we are able to tackle multiple priorities, and we're able to agree on where our bucks can have the biggest bang.

You then have fragmented research, where each researcher is interested in a different cancer. We try to bring them together across cancers so that they can learn from each other; even if the exact treatment might not work, a technique or an approach might work from one cancer to the other.

## What are the unique challenges for paediatric cancer compared to adult cancer?

**Jay:** It's a small patient population for each disease. Even if, altogether, there are a lot of patients, there are so many different diseases, and so you have difficulty finding treatments. You then have fragmented research, where each researcher is interested in a different cancer. We try to bring them together across cancers so that they can learn from each other; even if the exact treatment might not work, a technique or an approach might work from one cancer to the other.

**Liz:** Another unique challenge of childhood cancer is that, although everyone agrees that children are incredible, they usually don't have a fully formed voice in their own care. I think there's a balance with parents deciding for children. Depending on the age of the child, I think that can be a challenge for everyone.

A huge challenge in general is that children are developing quickly. The treatments that we are using to cure cancer in adults and children were mostly developed for adults. These treatments have pretty detrimental effects on their fast-growing bodies, and those side effects can stay with them for life. Brain tumours are a good example of where there's so much to consider when treating the child and their young developing brain that it makes it extremely challenging compared to treating adults because of those long-term side effects.

## How can researchers engage with organisations like yours better?

**Liz:** When they're invited to go to meetings organised by a foundation to speak, whether it's virtual or in person, really try to show up. It's this whole collaborative circle of allowing foundations to raise more money so that they can continue to pour money into research. And I would think that the researchers get a lot out of it. They get to share their science with a new audience of people, and who doesn't love to talk about what they're dedicating their lives to? If it's a scientific meeting, like our summits where we bring researchers together, they can really learn from others. There are so many opportunities to get involved with a foundation like ours. We have a lot of researchers who do fundraising with us in the month of September for Childhood Cancer Awareness month, and they get their whole lab involved. How empowering is that? So instead of just writing a grant and hoping the money comes in, we're allowing them to have their entire lab, their family members and their friends contribute to the work they're doing. I think that gives them much more ownership and pride in receiving that funding, knowing that they raised it.

**Jay:** The other way they can engage is when they share a success story that's connected to our work. Not only does it get our staff excited about what they're doing, but then we can share the stories with our donors and raise more money, and it just sort of feeds the whole cycle of research funding. So, we work hard to get those stories from researchers. There are some researchers that proactively reach out to us, but not nearly as many as we need.

… her story speaks to the successes that are not as final as a cure but show how meaningful it can still be to allow a family and a child to have hope, and to have significantly more time to do the things they want to do in their short lives.

## Are there any funded studies that you think have been particularly successful in moving into the clinical space?

**Liz:** To start with, Alex's life itself was a success story, as far as research goes. No, she wasn't cured, and it wasn't research we necessarily funded, but from the moment we enrolled her in a clinical trial, when we were told she was incurable, she lived 4.5 more years, and three of those years were very good years. They were some of the best years she had. She was 3 when we were told there was nothing more to be done, and she died at 8.5. She did so many things in those years, including starting her lemonade stand. So, I think her story speaks to the successes that are not as final as a cure but show how meaningful it can still be to allow a family and a child to have hope, and to have significantly more time to do the things they want to do in their short lives.

As far as our funding goes, there's one project out of the Children's Hospital of Philadelphia, with Dr Yael Mossé, who made a discovery about a mutation in some aggressive neuroblastomas, which happens to be the cancer that Alex had. They had treatments that existed for adult cancers with this mutation, and we gave her funding to test these treatments for children in a clinical trial. She had early successes with that trial where children, who were like Alex and were considered incurable, were going into remission very quickly and were staying there. We've met several of those families, so that's one that always stands out to me, because I see now how these children are growing up and living normal lives and doing all the things that I think Alex envisioned when she set up her lemonade stand and wanted to help other kids with cancer. A couple of the moms keep in touch with us, and I get to see what their kids are doing, so that to me shows you the progress. Then, kind of full circle, we learned that Alex's tumour had that mutation as well, so she could have been one of those kids, right? That speaks to how funding research and how work in the lab can actually come through and make a huge difference for children. It can cure kids who would not have been cured 15-20 years ago.

Another example is a researcher in Georgia, Dr Ted Johnson, who we gave an Early Career Award to, which, I believe, was his first grant. He was particularly interested in helping children who had relapsed and had hard-to-treat brain tumours. He has been running clinical trials, and families come to him who really don't have a lot of other options, and he is able to get them into his one of his various trials, treat them, give them more time and relieve their symptoms. In some cases, he's had children under his care for several years who continue to do well. For that particular diagnosis of paediatric brain tumour, I think that's really incredible. We know some of those families, and some of their children have passed away, but they had more time, and some of them are still doing well. The deficits they had from their brain tumours have actually improved with the treatment, so their quality of life is so much better. What they would view as a miracle – finding this doctor in Georgia who was able to offer them something that helped their child after so many things did not help them – to me, is what it's about. Actually knowing you're making a difference in a child's life; it's hard to even put into words what that would mean to those parents and to that family.

## It's amazing work and it must be incredibly rewarding. You've also branched out with your funding activities to set up broader initiatives, like The Childhood Cancer Data Lab. Why did you decide to set this up?

**Jay:** Like we said before, we're always looking for gaps that need to be filled, and we were trying to fill one of those gaps. Back in 2016, we went to our scientific advisors, and said, “We are looking to do something else with our investments. What are some areas that have need?”. In our mind, we had thought about big data, because back then it was all in the news and it was the buzzword topic. When we talked to our scientific advisors, they all brought up big data also, so we knew we were on to something. That's how we started it. A lot of times when you start something new, you go in with the mindset that this isn't going to change something overnight. It takes time. I think we opened in 2017, and so it's been 8 years that they've been doing the work, and it's evolved. What they do today wasn't what we envisioned they would be doing, but they do a lot of things that we never in our wildest dreams would have thought they would be doing. One of the big things they do is train people from childhood cancer labs on how to use data and how to set up experiments so they can get usable data, and then how to present the data. They're really into sharing data too, which, from a funder's perspective, makes total sense. If we're paying for this research, we want them to share their findings so it can help the broader cause.

## You have other grants that are aimed at helping families travel for appointments and care. Can you tell us a bit more about that?

**Liz:** Our Travel For Care programme was created in response to the fact that there are clinical trials open, but they're not open everywhere, as they are in specific locations. It is really uncomfortable, to say the least, that only families who could afford to travel would have access and the opportunity for that latest innovative treatment. So, the Travel For Care programme tries to at least solve some issues for families who want to go anywhere away from their home hospital to try an innovative therapy. These are almost always kids who have relapsed or are refractory and don't have any other options closer to home. If the treatment is successful, they may have to go back and forth for several months, and oftentimes there's a short window of opportunity and they may only have a week's notice to go. So there's pressure, and they can't save up or even borrow the money in that time. For a parent to have to make the decision that they can't go because they can't afford the plane ticket or the hotel or whatever is required, is unacceptable. That's why we created the Travel For Care programme, and it's a fairly simple solution to book airfare for families or to make sure they have a place to sleep, but it's had a huge impact.

**Jay:** It's not uncommon for these families to have never travelled before and they don't know what's involved with taking a flight. And sometimes the kids are very sick, and they need help getting through the airport. So, there's a lot of logistics and teaching going on also.

**Liz:** For research physicians who are planning clinical trials, it's really important for them to think about these things, because, at a minimum, if they want to accrue patients, they have to think about the logistical parts of what it would mean from the patient and the family's perspective to be able to participate. They need to think about what would be required to help them participate and proactively think about what resources are available. If they're being super proactive, they could think about finding the funds to make sure that they can help families enrol in the trial. They can also try to coordinate with patient's home hospitals for the blood work and those smaller things that happen in between. It would help everyone. It would help the families, which is the whole point, but it would also help their clinical trial, because it'll get much more participation.

## In the next 5-10 years, where do you hope childhood cancer research will have progressed to?

**Jay:** If you look at our goals, we want the cure rate to be more than 90% in the USA. And we want to be able to start figuring out how to raise the survival rate in middle- and low-income countries, where in some cases childhood cancer is probably back where the USA was in the 1970s. In order to do that, we need treatments that can be given without a lot of supportive care and hospital stays, because that's not an option in a lot of places in the world. That means getting away from chemotherapy and figuring out immunotherapy-type treatments that don't require hospitalisation.

Researchers who are in the lab and aren't MDs can find ways to hear those patient voices, whether it's through their collaborators, who are maybe paediatric oncologists, and just sitting down and talking with them or shadowing them at regular intervals.

## Why do you think researchers should value the voice of patients and advocates?

**Jay:** They're the ones that are going through the battle on the front line. They're the ones that know what happens when you're not sitting in an exam room, so they have to be listened to. You don't see the full impact on the kids until they get home: it could be a day later, could be a week later, after a treatment when they're feeling sick, can't get up, can't get out of bed, can't eat things.

**Liz:** Researchers who are in the lab and aren't MDs can find ways to hear those patient voices, whether it's through their collaborators, who are maybe paediatric oncologists, and just sitting down and talking with them or shadowing them at regular intervals. I think understanding and seeing a child who is fighting for their life, and hearing from the parents about all of the things that go along with that, is very motivating and plants in them the importance of their work. I do think it can shape the way you think about your research and what the goals of your research are and what that end goal should be, which is “how is this going to help a child who has cancer?”. That's what the research goal should be. It's good to learn things, but you should be learning things that will get you to that goal.

## Conclusions

Every child with cancer is unique, yet current treatments are far from tailored. Most children with cancer are given standard treatments that were designed for adults. These treatments can have extensive long-term side effects that are particularly dangerous in the developing body of a child (Chang et al., 2022). This highlights the ongoing struggle that some survivors of childhood cancer face, which is often underappreciated. The impact of cancer treatment throughout a survivor's life and potential approaches to mitigate these side effects is a key area of further research. One approach is to develop more targeted therapies. However, as most of these are initially being developed for adults, getting them approved for paediatric cohorts can be a long and arduous process. Clinical trials for childhood cancer are particularly difficult as their cancers are often rare and there are extra regulations for these trials. As Liz and Jay point out, these trials should be considerately planned to broaden patient access, which subsequently strengthens the research while making it more equitable.

The cure rate for childhood cancer in high-income countries is currently around 80%, and in low- and middle-income countries, where treatment access and comprehensive care is limited, it can be less than 30% (Lam et al., 2019). A core goal of ALSF is to increase cure rates globally, but this is only possible if novel, innovative treatments can be accessed and safely administered in these low- and middle-income countries. This means that, upon drug development, we need to consider the logistics of therapy transport, storage, administration and aftercare in the context of resource-poor settings.

Overall, ALSF is responsive to changes in the scientific landscape, as exemplified by their development of The Childhood Cancer Data Lab (Box 1), and is highly strategic in their funding approach. They maximise the impact of their funding by meaningfully engaging with patients and their families and considering patient populations beyond the USA to drive research that truly meets their needs.
